# Alone in the COVID‐19 lockdown: An exploratory study

**DOI:** 10.1111/asap.12317

**Published:** 2022-06-17

**Authors:** Rowena Leary, Kathryn Asbury

**Affiliations:** ^1^ Department of Education University of York, Heslington, York UK

## Abstract

Feelings of isolation have been prevalent worldwide since March 2020 due to COVID‐19 pandemic lockdowns. This has prompted increased concerns about loneliness and related mental health problems. During the first UK COVID‐19 lockdown, 71 participants were asked to share their high and low point stories from lockdown. These were analyzed using thematic analysis to explore how “aloneness” was experienced at this time. A deductive analyses supported three key facets of aloneness reported in the literature: *emotional loneliness*, *social loneliness*, and *existential loneliness*, as well as a more positive form of aloneness, *solitude*. An inductive analysis identified risk and protective factors for loneliness, comprising *worry*, *lockdown changes*, and *poor mental health*; and *social contact*, *emotional contact*, *stability* and *simple life*. The study highlights the importance of understanding how facets of aloneness interrelate, and how understanding risk and protective factors can help us to develop social and policy interventions to alleviate loneliness. In particular, solitude is proposed as a potential mechanism for alleviating loneliness, particularly existential loneliness, alongside more common social methods.

## INTRODUCTION

The COVID‐19 pandemic spread rapidly around the globe in 2020, resulting in many months of lockdowns and social distancing rules designed to protect public health. However, such measures caused widespread concern about mental health problems connected to isolation and loneliness. Studies conducted during the COVID‐19 pandemic indicate that loneliness has increased worldwide following global lockdowns (Killgore et al., 2020; ONS, 2020).

Even before the COVID‐19 pandemic, academics, health professionals and the general public were becoming increasingly worried about loneliness. It has been linked to an array of health problems, including depression, anxiety, cardiovascular issues and cognitive decline (Hawkley & Capitanio, 2015; van Winkel et al., 2017). Its severity has been likened to that of obesity and smoking (Lauder et al., 2006). The UK government had recognized loneliness as a major public health issue, setting out a strategy for addressing it and even appointing a Minister for Loneliness (Great Britain. Dept. for Digital, Culture, Media and Sport, 2018).

However, to address loneliness, the construct needs to be fully understood. Quantitative studies provide evidence for some of the types of loneliness experienced (e.g., Mund et al.^2019^; see van Tilburg, 2020,), but there are limitations in using closed response items when trying to understand such a complex phenomenon. Qualitative studies can generate richer and more nuanced data, suitable for exploring individual differences in experience, but these have tended to focus on older age groups (e.g., Dahlberg & McKee, 2014; Ost et al., 2020). There is little rich data available on lived experiences of loneliness in the general population, which could offer insight into the make‐up of this complex construct.

Further, in considering ways to address loneliness, almost all studies have focused on social mechanisms, without considering solitude: the enjoyable experience of being alone. Although there is a body of research on the benefits of solitude for broader wellbeing, the potential for solitude to play a role in improving lonely individuals’ wellbeing, alongside social mechanisms, is almost universally neglected.

This paper aims to address these gaps, using participants’ stories from the COVID‐19 pandemic to identify and analyze facets of aloneness, considering both loneliness and solitude.

## THE ALONENESS CONSTRUCT

It is well established that physical isolation does not equate to loneliness: it is possible to feel lonely in the presence of other people, or to enjoy being alone (e.g., Holt‐Lunstad, 2021). Loneliness has been defined as a separation between an individual's desired connections and their perceived actual connections (Peplau & Perlman, 1982). However, studies have further divided loneliness into subtypes, using a variety of descriptions.

This study considered three key elements of loneliness: social, emotional and existential, as well as solitude, forming a broad construct of “aloneness”.

### Social and emotional loneliness

Loneliness has commonly been separated into two facets: social and emotional. According to Weiss (1973), social loneliness relates to lacking social connections, such as friends, whilst emotional loneliness concerns missing close attachment relationships, such as a romantic partner or family. Weiss suggested that social and emotional loneliness correspond directly to social and emotional attachment needs and that making these relevant connections would resolve the loneliness experienced. Although Weiss acknowledged that other aspects of loneliness might exist, including loneliness related to existential concerns, he considered that these were rare. Weiss suggested that feelings of desperate loneliness are predominantly linked to emotional loneliness, so might be addressed through emotional connections.

Multiple studies have supported Weiss’ findings. For example, DiTommaso & Spinner (1997) conducted a study of students using the Social and Emotional Loneliness Scale for Adults. They found that social and emotional connections alleviated social and emotional loneliness, respectively, as suggested by Weiss. Other studies have considered risk factors associated with social and emotional loneliness, such as physical isolation resulting in reduced connections (e.g., the COVID‐19 pandemic: Labrague et al., 2021, and old age: Dahlburg & McKee, 2014). However, these studies have only used quantitative social and emotional measures, limiting their potential to identify further facets of loneliness beyond Weiss’ theory. Nevertheless, this body of research both supports the existence of social and emotional loneliness, and suggests that appropriate social interventions may be beneficial.

### Existential loneliness

More recent research has expanded this binary understanding of loneliness to add existential loneliness. This has been described as the source of both emotional and social loneliness (Mayers & Svartberg, 2001): a deeper form of loneliness, whereby the self is perceived as disconnected from others and the universe, together with feelings of emptiness, alienation and abandonment (Bolmsjö et al., 2019), and terror of death (Ettema et al., 2010; Mayers & Svartberg, 2001). Some researchers have described existential loneliness as having no permanent remedy (e.g., Mayers & Svartberg, 2001; van Tilburg, 2020).

Although research has provided some clarity on existential loneliness, clear gaps remain. A recent quantitative study (van Tilburg, 2020) considered existential loneliness alongside emotional and social loneliness. Tilburg found that, unlike social and emotional loneliness, existential loneliness does not result directly from a lack of desired connections, but relates to a deeper sense of disconnection from the world. Existential loneliness has also been linked to depression (Besharat et al., 2020), but although these studies noted overlaps between quantitative measures of depression and existential loneliness, it is unclear to what extent the constructs themselves overlap. Further, almost all studies on existential loneliness have focused on participants facing immediate existential issues: the ill, very old or dying (e.g., Ettema et al., 2010; Kitzmüller et al., 2018; Mayers & Svartberg, 2001). A handful of recent studies have found that existential loneliness is relevant to the general population (e.g., Helm et al., 2020; van Tilburg, 2020), but these appear to be limited to quantitative data. No extant studies, to our knowledge, have explored existential loneliness in the general population using a qualitative design.

Given that existential loneliness may be distinct from emotional and social loneliness, and that it currently has no known permanent remedy (van Tilburg, 2020), it seems evident that there is a real and urgent need for research exploring it. Unlike emotional and social loneliness, existential loneliness appears not to be resolvable through simple social solutions: rather, it may require a broader approach to interventions.

### Solitude

The positive aspect of aloneness, solitude, has been explored much less extensively than loneliness. Existing studies have examined positive experiences of being alone, including in childhood (Coplan et al., 2014; Galanaki, 2005) and old age (Pauly et al., 2017). For example, the benefits of solitude in childhood have been recognized as promoting the development of the self and improving general wellbeing (Galanaki, 2005). It is well accepted that choosing to be alone is an important factor in enjoying aloneness, whereas enforced isolation is much more likely to result in loneliness (Coplan et al., 2019; Chua & Koestner, 2008; Nguyen et al., 2018). Having an existing secure social network may also be important to enjoy solitude (Lay et al., 2019).

As in loneliness research, solitude studies have generally relied on quantitative data, for example, using the Preference for Solitude Scale (Burger, 1995). More recently, the Motivation for Solitude Scale (Thomas & Azmitia, 2019) recognized the importance of understanding why people choose to be alone. This indicates the need to understand why and how solitude occurs, and to contrast this with circumstances resulting in loneliness, developing current research which has tended to focus on loneliness and solitude separately.

Existing studies are ambivalent about the meaning of solitude. Some acknowledge that, like loneliness, solitude has a variety of forms and degrees: solitude can be experienced in the presence of others, such as in a busy cafe (e.g., Lay et al., 2019), or even with an intimate other, seeking separation from the world together (Long & Averill, 2003). Many studies define solitude as being alone voluntarily (e.g., Nguyen et al., 2018; Pauly et al., 2017).

Exploring experiences of solitude alongside loneliness, using rich narrative data, will enable comparisons to be drawn between the development of both, and to explore whether solitude may support individual wellbeing to prevent loneliness, alongside social mechanisms.

## THE ROLE OF SOCIAL MEDIA

Social media has become a key tool for virtual communication in lockdown. However, it is known that social media connections do not compensate for lacking physical relationships (Nowland et al., 2018; Stepanikova et al., 2010). Although social media may benefit those who do not report being lonely, it may be detrimental to the wellbeing of those who are very lonely (López et al., 2019). Negative outcomes have been particularly evident when social media is used extensively (Bekalu et al., 2019; Wang et al., 2018). Consequently, although social media may be a useful mechanism to enhance and maintain existing connections, these studies suggest that social media may not be particularly useful to combat loneliness for those already suffering.

## NARRATIVE IDENTITY THEORY

The theoretical framework for gathering data in this study was narrative identity. Narrative identity is the story of the self, made up of one's reconstructed past, perceived present and imagined future (Adler et al., 2017; McAdams, 2001). Narrative identity thus concerns how an individual's subjective experiences influence their self‐ perception. An individual's narrative identity is evident through personal recollections of key life events; for example, through “storytelling” within the Life Story Interview developed by McAdams (2001), where individuals recount a high point and low point story, among others. Narrative identity storytelling methods were chosen to enable the collection of data that could facilitate a deep understanding of how aloneness constructs exist within individuals’ subjective experiences.

### Aim

This study aimed to identify the facets of aloneness experienced by participants during the first UK COVID‐19 lockdown, gain insight into how they were experienced, and explore what helped to protect some participants against loneliness. We asked two research questions:

1. Is there evidence of distinct facets of aloneness in participants’ high and low point stories during the first UK COVID‐19 lockdown?
2. What risk and protective factors in relation to loneliness were evident in their stories?

## METHODS

### Participants

Seventy‐one participants aged 17–73 (median age: 39; 51 females, 19 males, one other) completed the survey. Participants were from the United Kingdom (64), New Zealand (2), United States (2), China (1), Russia (1) and France (1). Although not all participants were from the UK and international participants may have experienced lockdowns slightly differently, this is a study of aloneness constructs rather than the COVID‐19 pandemic, so any differences in experiences do not impact on the study's validity. Nevertheless, varying local COVID‐19 lockdowns may have affected participants’ individual experiences of aloneness, as noted in the limitations section.

Participants provided some data about their occupational status, reporting a range of titles including lawyer, postal worker, athletics coach, nurse, teacher, research assistant, bank clerk and student. Most participants reported being affected by COVID in their occupations, particularly the requirement to work from home.

These data provide a flavor of the participants’ backgrounds, but they have not been analyzed quantitatively as the purpose of this study was to explore participants’ stories and the themes identified within these narratives. Further background information about participants (such as socio‐economic status or race/ ethnic background) were not gathered.

### Measures

Participants provided demographic information and answered free response questions about lockdown.

These questions were based on items from McAdams’ Life Story Interview (McAdams, 2001). Participants were asked to describe their high and low points during lockdown (later known in the UK as the first COVID‐19 lockdown between March 26, 2020 and June 1, 2020), with the purpose of exploring experiences of aloneness that were shared spontaneously in the stories they selected and the way they told them. On average, participants provided stories of 101 words for high point stories (range: 5–393) and 110 words for low point stories (range: 1–623). Participants were asked to write stories according to the following prompts:

*Please identify a scene, episode, or moment during the COVID‐19 lockdown that stands out as an especially positive experience. This might be the high point scene of the lockdown, or else an especially happy or joyous moment during this difficult time. Please describe this high point scene in detail. What happened, when and where, who was involved, and what were you thinking and feeling? Also, please say why you think this particular moment was so good and what the scene may say about who you are as a person*.

*Please identify a scene, episode or moment during the COVID‐19 lockdown that stands out as a low point. This might entail loneliness, sadness or fear, or some other negative emotional experience. Please describe this low point scene in as much detail as possible. What happened in the event, where and when, who was involved, and what were you thinking and feeling? Also, please say why you think this particular moment was so bad and what the scene may say about you or your life*.

It should be emphasized that participants were not asked specifically to write stories about being alone, allowing for spontaneous accounts of aloneness to be identified within participants’ stories.

Participants were also asked to answer a free response question about their experiences of social media during lockdown, as follows:

*Please describe how social media has affected your experience of the Covid‐19 lockdown*.

Quantitative loneliness data were also collected via a single item which asked whether participants recalled feeling lonely during lockdown, using a four‐point Likert scale with options of “never”, “rarely”, “sometimes” and “often”. This single‐item question aimed to gain a broad‐brush understanding of participants’ own views of their loneliness levels during lockdown. The intention of this question was to gain an understanding of participants’ recollected, self‐perceived loneliness levels during lockdown, as context for their narrative descriptions. A simple, single‐item question was therefore chosen above a standardized multi‐item loneliness measure in this context. However, as explained in the limitations section, this measure should not be taken as a robust or validated measure of participants’ loneliness levels. Nevertheless, the single measure item provided participants with a simple question about their subjective impression of loneliness, enabling a straightforward answer compared with multiple item measures (which although robust, are less direct because they break down “loneliness” into academically determined components).

Participants’ responses to this measure, along with gender and age, are stated with their quotations in the results to provide context for their comments.

### Procedure

The data were collected as part of a wider project on loneliness during the first UK lockdown. Participants completed an online survey via Qualtrics, which was considered suitable because a large proportion of the global population was at home during this time with technology access.

The survey was published on June 3, 2020 and remained open for 4 weeks. It was shared via social media (Facebook and Twitter).

### Analysis

Data were anonymized by replacing identifiable names or locations with a general term prior to analysis.

Thematic analysis was used because it enables patterns of meaning to be identified across the data to address the research questions (Boyatzis, 1998; Braun & Clarke, 2013). A two‐part approach was taken to address each research question. Participants’ responses to all three open questions (high point, low point and social media use) were analyzed for both rounds of analysis (although for research question two, only “never” and “often” lonely participants’ stories were considered, as described below).

To address the first research question, a deductive approach was taken. This allowed for a focus on the facets of aloneness identified in the literature, and on whether and how they were experienced by the participants. Although this analysis was primarily deductive we also remained open to identifying new or different facets of aloneness in the data.

The data were read and re‐read by the first author for initial familiarization, then initial codes were generated and then collated and reviewed. Overlapping codes were collapsed into a single code, and appropriate labels were applied to the final codes. The codes were clustered into themes, and appropriate labels applied to the themes. These labels were guided by the literature and by the deductive approach we took. The themes identified were re‐checked against the data to ensure they reflected the participants’ stories accurately, and against the research question to check it was being addressed directly.

The second research question was addressed by coding and analyzing participants’ high and low point stories, as well as their responses to the social media question. For this analysis, an inductive and primarily semantic, essentialist approach was taken, allowing codes to be generated that reflected participants’ subjective experiences. The codes were clustered together into themes, and labels applied that described the patterns of risk and protective factors described by participants across the data. The themes were re‐checked against the data to ensure they corresponded with participants’ narratives accurately.

Although the full data set was considered to identify risk and protective factors, the second research question was addressed by comparing participants who reported being “often” and “never” lonely in lockdown. Selecting these groups only was appropriate as this provided focus on factors that helped or worsened subjective loneliness.

### Ethical considerations

The survey included a consent form on its first page containing information about questions participants would be asked and details of three support organizations, as well as confirmation that data would be anonymous. Participants had to confirm acceptance of this information before commencing the survey. Additionally, participants were reminded of the support organizations’ details on the survey's final page. The survey was subsequently approved by the Ethics Committee at the authors’ institution.

## RESULTS

### Reported loneliness

**FIGURE 1:**
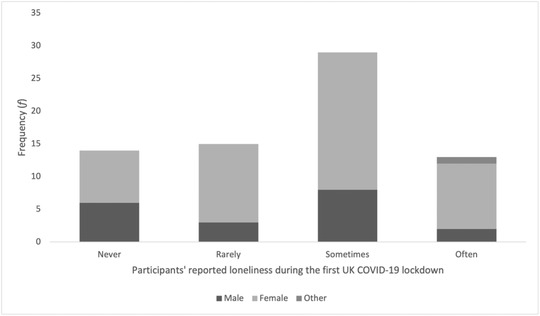
Participants’ reported loneliness

Participants’ responses to the question, “Have you experienced feelings of loneliness during the COVID‐19 lockdown?” are summarized in Figure 1. Notably, almost a third of males (six of 19 males) reported “never” feeling lonely in lockdown, but only 16% of females (eight out of 51 females) reported this. These males’ low point stories appeared to support the quantitative measure (they did not contain loneliness themes), but this may reflect a gender difference in how loneliness is perceived or reported, rather than in loneliness itself. As noted in the methods section, this single‐item measure was intended to provide a simple measure of participants’ recollected, self‐perceived levels of loneliness, alongside their stories, and should not therefore be taken as a reliable or validated measure of the loneliness that individuals experienced.

RQ1: Is there evidence of distinct facets of aloneness in participants’ high and low point stories during the first UK COVID‐19 lockdown?

Four aloneness themes were identified. In low point stories these were (1) *emotional loneliness*, (2) *social loneliness*, and (3) *existential loneliness*; in high point stories the only aloneness theme was *solitude*. Although there were areas of overlap between all aloneness themes, suggesting a common overall construct of aloneness, there were clear indications that these four facets of aloneness were distinct constructs with different characteristics, as described below. The aloneness themes identified mapped onto the facets identified in the literature, and no new facets were identified.

The aloneness themes were identified through clustering codes together that described the data closely. For example, *emotional loneliness* included the codes of *missing family* ‐ as indicated, for instance, by “*missing my parents – my Dad's 80th birthday is tomorrow so this weekend should have been a big family celebration*…” (female, 43, never lonely) – and *lack of romantic partner*. Similarly, *social loneliness* included the codes of *lack of organized contact*; for example, “*I enjoy football… At the point of lockdown, this team activity was cancelled… I suddenly found I was unable to engage with fitness activities in the same way*.” (male, 42, sometimes lonely), *lack of colleague contact*, and *missing friends*. Although missing others indicated the possibility of a relationship, the fact that desire was unfulfilled in the reported memory was interpreted as indicative of loneliness (Peplau & Perlman, 1982).

Existential loneliness was identified through codes that described feeling disconnected and fear of being alone, such as “This was a bad time as I realized that I need people and I am frightened to die alone. My life before lockdown was good and now I realize that I am very alone.” (female, 60, sometimes lonely). Further codes clustered into this theme included withdrawal and feeling lonely in the presence of others.

Solitude was identified via codes that described a joy or appreciation for solitary moments, such as exercise, personal achievements and nature; for example, “I am happy to be alone, and am very relaxed when out in nature and exercising, I can escape from stress by appreciating other things happening around me.” (female, 23, never lonely).

### Emotional loneliness

This theme comprised participants’ stories about missing close personal connections: family or romantic partners.

Although there were examples of *emotional loneliness* in participants’ low point stories, the theme was notably less prevalent than *social loneliness*, reflecting that many people spent lockdown with partners or family.

Reports of *emotional loneliness* linked to the absence of a romantic partner suggested that the isolation of lockdown had brought their loneliness to the fore. For example:

I have my family, friends and neighbors who were all very helpful but I missed not having a special someone at home… I miss having a life partner to help me in times of stress to see the bigger picture and talk through my fears, someone who can shoulder some of the burden. It is quite a heavy one to carry on my own… (female, 45, sometimes lonely)

This narrative suggests that the lack of a partner has left a gap that cannot be filled by wider family or friends, reflecting that *emotional loneliness* is a separate construct from *social loneliness*. However, participants also indicated that *emotional loneliness* was connected to other forms of loneliness: one participant (female, 27, sometimes lonely) described her feelings following a break‐up:

I went for a walk by myself and felt very low and lonely. It made me feel like I had no‐one in my life, no social connections and no support system. I felt very alone… at this point in time it felt like I would never have friends or be able to meet anyone… I feel that I need physical contact such as hugs to feel connected to another person…

This indicates that *emotional loneliness* may enhance feelings of *social loneliness*, spiraling into despair and *existential loneliness*.

This participant's desire for physical contact was also common in stories about missing family in lockdown, even for participants who reported “never” being lonely. For example, one participant described their father's 80th birthday celebration, to be celebrated via Zoom, when “*I just want to give [my parents] a hug.”* (female, 43, never lonely). This indicates that virtual presence, for this participant, was not enough to fulfil their desired connection to their family. Another, whose mother had become unwell, explained that “*I don't often feel lonely ‐ and in fact love living alone ‐ but I felt particularly conscious that there wasn't anyone there to give me a hug and missed the possibility of human touch.”* (female, 40, never lonely). Although these examples indicate that physical touch with close connections may be important to avert feelings of loneliness, these participants did not describe despairing feelings evident in romantic *emotional loneliness* stories.

These stories indicate that *emotional loneliness* itself is multi‐layered. It may occur alongside *social loneliness*, but appears to be distinct from this construct. Missing family during lockdown, particularly at emotional times of celebration or stress, appeared to relate directly to the need for physical contact. In contrast, participants who did not have a romantic partner appeared to be at risk of a deeper spiral towards *existential loneliness*.

### Social loneliness

*Social loneliness* was a very common theme in participants’ low point stories, reflecting COVID‐19 lockdown restrictions against socializing outside households. Several layers of *social loneliness* were evident.

Some participants’ experiences of social loneliness were closely linked to emotional loneliness. For example: “Despite my partner being there I felt alone and fed up and desperately unhappy… I realize that my close friends are my family as I'm not particularly close to my parents or siblings…” (female, 41, rarely lonely). This demonstrates that emotional and social loneliness may overlap, depending on the individual's perception of who constitutes their “family” and their “friends”. For this participant, connections with friends appeared to carry stronger emotional weight than those with family. In this case, the distinction between social and emotional loneliness has become blurred.

However, generally participants appeared to recognize that *social loneliness* would be resolved by reinstating social connections. In contrast with stories of *emotional loneliness*, participants describing *social loneliness* tended to blame lockdown for their loneliness, rather than the feeling manifesting internally as a personal fault and spiraling into deep, *existential loneliness*. This resulted in milder feelings of frustration or sadness at the lockdown situation, particularly for participants who felt they were at particularly “social” stages of life, such as starting university: “*This is the point in my life I feel like I want to be away from home and being spontaneous and messing around with my friends, which I can't do when it's just me in my room alone…”* (female, 20, often lonely) or retirement: *We are an active and socially gregarious couple… life started to look pretty empty very suddenly*… (male, 68, sometimes lonely). One participant described severe *social loneliness*, but they still related this directly to their need for social connection: “*Sometimes the loneliness and anxiety becomes unbearable… this reflects my reliance on my friends to socialise with…”* (female, 17, often lonely).

Consequently, there appears to be a distinction between the facets of *emotional* and *social loneliness* in participants’ stories, in how the constructs are experienced, and the ways that they might develop or worsen. It appears that *social loneliness* may be less likely than *emotional loneliness* to be internalized, and may carry less risk of developing into *existential loneliness*.

### Existential loneliness

The theme of *existential loneliness* was apparent in participants’ low point stories, both independently and connected to *emotional* and *social loneliness*. As noted above, it appears that experiences of *emotional loneliness* and, occasionally, *social loneliness* were at risk of being followed by feelings of *existential loneliness*, implying that *existential loneliness* may underlie, or be a consequence of, these well‐established facets of loneliness.

Examples of *existential loneliness* were distinguishable from *emotional* and *social loneliness* because they did not necessarily describe the lack of a specific relationship; rather, negative experiences (including *emotional* or *social loneliness*) appeared to trigger a more general, inward feeling of isolation or disconnection.

A key aspect of this theme was participants’ descriptions of feeling generally lonely or disconnected despite the presence of others. For example: “*I was stood in the kitchen and I remember thinking that although I have my kids around me and a partner I feel like I'm on my own. I think this sums up my life, very little support from my partner, feeling like I have to get through things on my own.”* (female, 40, sometimes lonely). This suggests that lockdown may have exacerbated a pre‐existing lack of emotional connection, resulting in greater feelings of *existential loneliness*. Some participants described specific reasons for experiencing disconnection with loved ones; for example, differences in worry about lockdown rules: “*I called my parents and was disappointed and scared that they were not yet taking social distancing seriously… My feelings were hurt when they brushed off my concerns ‐ it made me feel more isolated.”* (female, 26, sometimes lonely). These examples indicate how difficulties in relationships might develop beyond a desire for specific emotional or social connections, resulting in more general feelings of disconnection associated with *existential loneliness*.

Another feature of *existential loneliness* was its link with *poor mental health*. For example, some participants expressed a desire to withdraw (also a symptom of depression), despite acknowledging that this was counter‐intuitive for their loneliness: “*I tend to hate being alone but at the same time during lockdown at my lowest I wanted to be alone.”* (female, 19, sometimes lonely). Other participants described fear of being alone; this is a particularly debilitating characteristic of *existential loneliness*. For example, “*This was a bad time as I realized that I need people and I am frightened to die alone. My life before lockdown was good and now I realize that I am very alone.”* (female, 60, sometimes lonely). This demonstrates how, for some, feelings of forced isolation and a lack of autonomy in lockdown spiraled into feelings of fear of death associated with *existential loneliness*.

These examples demonstrate that *existential loneliness* is distinct from *emotional* and *social loneliness*. It appears to affect individuals more deeply, and is more closely connected to *poor mental health*. However, *social* or *emotional loneliness* may be at the root of *existential loneliness* in some stories.

### Solitude

As well as the negative “loneliness” elements of the aloneness construct, a positive theme of being alone, *solitude*, was identified in participants’ high point stories. Solitude stories tended to relate to activities participants had chosen to do alone, indicating that autonomy was an important aspect of the theme.

Generally, *solitude* did not overlap with loneliness themes, but rather, countered them. Nature was commonly referred to in stories of *solitude*: being alone appeared to enable participants to appreciate the beauty of the outdoors, often whilst exercising. For example: “*I went for a walk into the field. The weather was sunny and the trees and flowers were in full bloom and I finally got some time to myself away from my family.”* (female, 26, sometimes lonely). This participant specifically noted that being alone, away from their lockdown household, was important: *solitude* appeared to counter their negative feelings. This is supported by other participants’ stories about wellbeing benefits from nature and exercise:

On one particular run I came across eight Kites (birds) circling a field, which was a really positive experience. I was on my own and I was feeling very elated and lucky to witness the birds so close to my head… I am happy to be alone, and am very relaxed when out in nature and exercising, I can escape from stress by appreciating other things happening around me. (female, 23, never lonely)

It appears from these participants’ descriptions that being alone is an important element of fully appreciating and connecting with nature, to counter negative feelings.

For some participants, exercising alone in itself was a high point, as this participant (other, 30, often lonely) described:

It was so good to be skating, it was exhilarating… It was a good feeling to just do something by myself and for myself for no other reason than it made me happy. I didn't take any photos or post about it on social media, I liked that it was just my thing… I think it made me feel like a whole, individual person who has a life and interests… it helped me express my personality… When I saw this question, I started thinking through social events I have had during lockdown as I am quite a sociable person… but going skating by myself that one time stands out as a time I was really joyful.

This shows how being immersed in a solitary activity by choice can be joyful, enabling expressions of self and identity, and increasing autonomy, and in turn, *stability*. It is notable that this participant says that they consider themselves a sociable person, but still chose a solitary activity for their high point. Their story contrasts with low point stories of participants experiencing *existential loneliness*, who expressed a fear of being alone, demonstrating the extremes of the aloneness construct.

Activities requiring concentration, such as work, or achieving personal goals, were also connected to high point stories of *solitude* in its wider sense: focusing on an individual activity regardless of the presence or absence of others. This linked to feelings of being in control, and personal fulfilment. For example:

I bought a flat pack desk… I tackled it myself one evening ‐ with a glass or two of wine ‐ and made a pretty good job of it. I felt like I had really proved something to myself and that I was stronger, more resilient and more self sufficient than I sometimes give myself credit for. (female, 40, never lonely).

As in the examples relating to nature and exercise, this high point story indicates the possibility of self‐development that activities in *solitude* can offer.

Similarly, others described the chance to focus more fully on work in *solitude*, without the distraction of the social workplace:

I stayed in my room without any physical contact with my friends and just focused on my work without any interruptions. It makes me more concentrate [sic]. I think it is a good moment for us to stay calm and relax. (female, 22, sometimes lonely)

This illustrates how aloneness can be experienced positively, and again contrasts with those who described being alone with fear, or as an example of loneliness, within their low point story. Being able to feel that *solitude* is positive may result from individual differences, but supporting those suffering from loneliness to appreciate *solitude*’s benefits may help to improve their wellbeing.

Some high point stories containing the theme of *solitude* overlapped with social themes, such as *social contact* or *emotional contact* (themes which are outlined in the second research question). For example, one participant described the pleasure of enjoying coffee in a cafe: “*I enjoy the atmosphere because I sit alone and not be alone…”* (female, 26, sometimes lonely). This suggests that being in *solitude* may not necessarily always involve physical isolation: sitting in a cafe alone may provide the benefits of solitary reflection or concentration without feeling completely alone. *Solitude*, like loneliness, therefore appears to be multi‐faceted, and may answer individual needs in a variety of ways, including existing alongside *social contact*.

This was further exemplified by high point stories about deep reflection in nature or exercise (i.e., elements of solitude) that were experienced with one family member or a partner, and were thus overarchingly linked to *emotional contact* (outlined in the second research question). For example, one participant described running with his son:

I could feel the warmth of the sun on my skin, and could smell wild garlic in the woods which also triggered happy memories. I felt completely at peace. I rationalized this with the fact that lockdown had slowed down my pace of life. (male, 45, rarely lonely)

This story demonstrates how lockdown provided some individuals with an appreciation of time and the *simple life*; these were generally characteristic of the theme of *solitude* as they would not appear to require company, but were also enjoyed with one other person. This was also evident in experiences shared with partners; for example:

A 5 h walk from home with partner and dogs. Loved the quietness… it was lovely being able to enjoy where we live without the crowds… Confirms my knowledge about myself that I'm at my happiest when walking in countryside, away from the world, with the ones I love, that is, partner and dogs. (female, 43, rarely lonely)

This participant specifically notes the importance of quietness, being away from others, and enjoying the countryside: all elements of *solitude*. However, they also had their partner with them, resulting in the joy of being alone, seen in other participants’ *solitude* high points, being replaced with being “with the ones [they] love” (*emotional contact*).

Finally, there were some examples of low point loneliness stories overlapping with *solitude*; for example, “the sea… looked very lovely. It gave me mixed feelings. It was beautiful and peaceful but sad because I was alone on the empty beach due to the lockdown.” (female, 73, sometimes lonely).

These stories demonstrate that *solitude*, like loneliness, is multifaceted and may co‐exist alongside positive *social* or *emotional contact* themes, as well as falling within the broad construct of aloneness alongside facets of loneliness. Whilst *solitude* with *emotional contact* requires a close family or romantic connection, *solitude* with *social contact* may be beneficial for individuals who find *solitude* difficult to enjoy in physical isolation.

In response to the first research question, we found that all the facets of aloneness in the literature were identified in our data. This adds depth to our understanding of how those facets can be experienced by individuals, and the possibility of a multi‐faceted aloneness construct comprising solitude and existential loneliness, as well as emotional and social loneliness.

RQ2: What risks and protective factors in relation to loneliness were evident in the stories?

**FIGURE 2:**
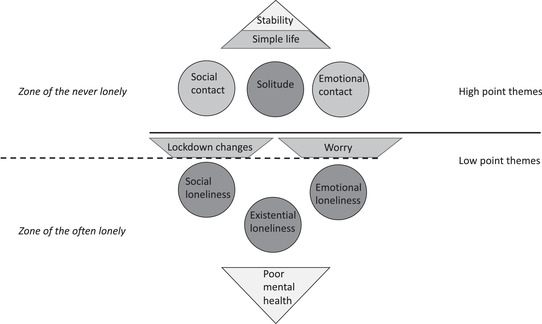
Thematic summary of facets of aloneness and related constructs *Note*. 1. Distinct facets of aloneness (RQ1) are shaded dark grey. 2. Risk and protective factors (RQ2) include the themes of *simple life, lockdown changes* and *worry*, as well as factors connected to the aloneness facets set out in Figure 2.

Using an inductive approach, we identified seven themes representing risk and protective factors for the facets of loneliness described above. These can be seen in Figure 2.

When stories of participants reporting “never” being lonely in lockdown were compared with “often” lonely participants, it became clear that the “never” lonely group had described a narrower range of low point themes, but a wider range of high point themes. The groups were of comparative sizes (for “often” lonely participants, *n = 13*; for “never” lonely, *n = 14*). Consequently, exploring the differences between these groups’ reported experiences provides an insight into potential risk and protective factors in relation to loneliness. As noted previously, this was a simple, single‐item measure that gave participants an opportunity to identify their self‐perceived loneliness level during lockdown.

### Summary of themes

Five themes (including the aloneness theme of *solitude*) were identified in participants’ high point stories: (1) *solitude*, (2) *emotional contact*, (3) *social contact*, (4) *simple life*, and (5) *stability*. Six themes (including the aloneness themes of *emotional, social* and *existential loneliness*) were identified in the low point stories: (1) *emotional loneliness*, (2) *social loneliness*, (3) *existential loneliness*, (4) *worry*, (5) *poor mental health*, and (6) *lockdown changes* (see Figure 2).

The high point themes of *solitude*, *emotional contact* and *social contact* appeared to underpin participants’ wellbeing. Where they felt happy in their “aloneness” status (whether this meant being in solitude or with others, according to individual preference), they were able to appreciate *simplicity*, such as the joy of nature or spending time at home with family; these settled scenes allowed participants to enjoy a sense of *stability* and heightened wellbeing.

Conversely, the low point themes of *emotional loneliness* and *social loneliness* appeared, at times, to spiral into *existential loneliness*, and further into worsening *poor mental health* (as described in detail below). However, low point themes of *worry* and *lockdown changes*, which appeared less detrimental to mental health, were also identified. These were common in low point stories of “never” lonely participants.

The range of themes identified in the stories of “often” and “never” lonely participants suggest that the themes can be divided into two zones, as shown at Figure 2: “often” lonely participants’ low point stories contained a wide range of themes, including all three types of loneliness and *poor mental health*, but their high point stories contained limited themes; on the other hand, “never” lonely participants’ low point stories tended to be limited to themes of *worry* or *lockdown changes*, and their high point stories demonstrated a wide range of high point themes.

### Risk factors

The low‐point stories of participants who reported being “never” lonely commonly included the themes of *lockdown changes* and *worry* (particularly worry for loved ones). For example: “*I would wake up in the morning and for the first few seconds, I would say to myself is this all a dream? Then you realise it's not!”* (male, 59, never lonely). However, the worry within such stories did not appear to be internalized by “never” lonely participants beyond a low level of concern: these themes therefore generally appeared to be distinct from stories containing the theme of *poor mental health*. One participant did report a negative effect of being alone on their mental health ‐ but, notably, they also explained that they had taken action to alleviate these feelings: “*… I have… moved to my parents house to solve that problem… I was talking daily to friends and family over video calls at this time”* (female, 23, never lonely). This participant did not therefore become stuck in loneliness: they recognized and removed the risk of loneliness developing.

This contrasts with the stories of those reporting “often” being lonely. Although they too told low point stories containing themes such as *lockdown changes* and *worry*, the full range of other low point themes were also evident in their stories, including *emotional loneliness* and *social loneliness*, which at times spiraled into *existential loneliness* and *poor mental health*. For example: “*I was worried about being isolated at home with nothing to do and no one for company. I was quite worried about being alone for some time…”* (female, 39, often lonely). This indicates that the “often” lonely may have had pre‐existing negative associations with being alone before lockdown, and consequently that they may have been affected more deeply by *lockdown changes* and *worry* than the “never” lonely group, and more likely to fall quickly into a sense of panic, fear, existential loneliness and poor mental health.

Another participant's story exemplified this point, suggesting that their lack of contact with others, that is, the social or emotional loneliness resulting from physical isolation – was having a deep impact on their mental health and self‐care:

There's no motivation to do anything and the things that need doing seem overwhelming. It's very difficult to motivate myself to do exercise as well even though I'm usually a sporty person. This reflects my reliance on my friends to socialize with… (female, 17, often lonely)

Here, there is an implication of powerlessness and feeling overwhelmed resulting from *social loneliness* in this story, which differs from the “never” lonely participant described above, who took action to prevent their anxiety stemming from being alone spiraling.

These reactions may be a result of individual circumstances and differences, or may relate to pre‐existing *poor mental health*. For example, one participant described a supermarket trip during lockdown:

Going shopping with my boy at [the supermarket] and being completely stressed… I came home and cried because I was so stressed and then anxious that I could have caught the virus because of people who don't follow rules. This moment shows how much my anxiety and depression was going to rule my life over the next few weeks. (female, 49, often lonely)

The implication that this participant felt powerless – “ruled by” their depression and anxiety ‐ which had been triggered by worry about COVID‐19, contrasts with the participants in the “never” lonely group, who either described lockdown more factually, or took steps to alleviate their situation (thus taking more control).

“Often” lonely participants also appeared to be more negatively affected by lockdown social media use compared with “never” lonely participants. One participant commented that “*I have found some social media difficult as it reminds me that I am alone”* (female, 39, often lonely). For this participant, social media was an acute reminder of their loneliness in real life, worsening their sense of isolation through comparisons with others. This links to the social and emotional loneliness that some participants reported in low point stories, resulting in worsened wellbeing.

Additionally, some participants reported social media impacting negatively on their lockdown experience due to hype, negativity or because it wasted time; for example, “*I hate it, I feel like it has made everything feel worse for me. It's not like actual socializing at all, it's just tiring and upsetting. I either feel left out of things or annoyed at people not following guidance*.” (other, 30, often lonely). This demonstrates the participant's impression of negativity in social media, implying that seeing posts of others’ actions can further isolate individuals through a lack of any real social or emotional connection.

That “often” lonely participants reported a broad range of low point themes indicates that external factors (such as COVID‐19 worries) may constitute risk factors for loneliness, triggering, or co‐existing with, deeper emotional difficulties such as *existential loneliness* and *poor mental health*. Such participants generally appeared to feel powerless to act against these consequences. On the other hand, the “never” lonely group appeared to have mechanisms to protect themselves against loneliness occurring, despite also being in lockdown: this enabled them to remain predominantly in the “never lonely zone” (see Figure 2), so they did not descend into existential loneliness or poor mental health. Analysis of these groups’ high point story themes may offer insight into who might be most at risk of developing loneliness, and what protective factors may alleviate or prevent it.

### Protective factors

Participants reporting “never” feeling lonely in lockdown told stories containing a wide range of high point themes. These included *emotional contact*, such as enjoying time with family: “*The whole lockdown was quite relaxing for me. I enjoyed more time with the family, cooking and walking the dogs*.” (male, 46, never lonely), or a partner:

My fiancé and I had a “date”, in which she prepared a three course meal and we both wore our nicest clothes and acted as though we were at a restaurant… It made me feel extremely grateful ‐ not just for the meal, but to be able to share my life with someone so wonderful. It also reminded me that so long as I have her in my life, my life will have meaning and purpose. (male, 28, never lonely).

These examples are characteristic of individuals with secure, emotionally‐close connections which offered them a source of support, helping to prevent loneliness. However, “never” lonely participants also reported examples of a variety of *social contact* high points, including “*speaking to family a lot more thanks to Zoom”* (male, 59, never lonely), and helping others: “*Helping a friend with his medicine, groceries and other shopping needs since he has mobility issues. I believe humanity needs to work together to combat any global issues”* (male, 52, never lonely), as well as high points of *solitude*, such as building a flat‐pack desk or appreciating nature (both described under *Solitude* above), and *simple life*: for example,

The whole lock down was quite relaxing for me. I enjoyed more time with the family, cooking and walking the dogs. It was nice not to have the constant pressures of work and everyday life. (male, 46, never lonely).

This wide range of high point themes indicates that “never” lonely participants were not solely reliant on social or emotional contact for their wellbeing, but appreciated a variety of experiences, including solitary ones, resulting in increased *stability*. However, the direction of effects between the range of experiences enjoyed by these participants and their lack of loneliness is not certain: those who have not experienced loneliness and who have strong support systems may find it easier to enjoy solitude as well as social high points.

This trend was supported by “often” lonely participants, who told stories containing a narrower range of high point themes, generally relating to social experiences. Some stories indicated that isolated participants treasured rare moments spent with others; for example:

My sister came to my house to bring me an Easter egg on her way to work which was a complete surprise and very thoughtful of her. It was a long bank holiday weekend so I was not working and was feeling quite lonely. It was lovely to see someone in the flesh and have a face to face conversation. It was also nice knowing someone was thinking about me. (female, 39, often lonely)

This indicates that brief connections can lift those who feel lonely, particularly where the connection is meaningful: this participant knew that her sister had been thinking about her, providing a sense of emotional connection as well as a brief physical presence.

Similarly, examples of *emotional contact* through close family experiences were important high point themes for lonely participants:

[My son] requested a bike ride… I chose to run, and we went off side by side and just chatted… I was Mum, doing what Mum loves; running alongside one of my favorite people who was also doing what he loves, talking animatedly… (female, 37, often lonely)

This participant's sense of identity during time spent with her son is clearly strong: this meaningful aspect of the emotional connection resonated with her as a high point.

Social media also provided some participants with protection against loneliness, particularly social and emotional loneliness. As one participant explained,

I really enjoyed having a Zoom call with my flatmates from university where we were putting random backgrounds behind our faces as a joke. I felt way less lonely and it actually felt like something that I wouldn't have had if lockdown hadn't happened. All of my Zoom/FaceTimes with friends or family have been really nice. (female, 20, often lonely)

This indicates that for some “often” lonely participants, social media was useful to maintain pre‐existing meaningful connections, connecting to the theme of emotional contact.

The fact that “often” lonely participants’ high point stories tended to contain themes narrowly linked to social or emotional contact stood out in analysis, in comparison with the wide range of themes found in “never” lonely participants’ stories. It appears that the “often” lonely participants’ limited social connections were not sufficient to enable them to move out of the “zone of the often lonely” (see Figure 2) altogether.

However, although “often” lonely participants generally cited social stories for their high points, one participant (other, 30, often lonely) in this group chose a solitude high point about skating alone, providing a detailed insight into her thought process in selecting this story above social experiences (as described above under *Solitude*). This indicates that solitary experiences can provide joy, including for those who are lonely. However, solitude was not otherwise a theme selected by “often” lonely participants for their high points.

Lonely individuals may feel unable to “enjoy” being alone, or be reluctant to do so ‐ but the fact that “never” lonely individuals selected a wider variety of high points, including solitude, suggests that enjoying solitude as well as social connections may offer protection against loneliness.

## DISCUSSION

The COVID‐19 pandemic lockdown provided a useful context to examine facets of aloneness experienced in the general population, and to highlight the distinction between them. A qualitative approach made it possible to explore whether the facets of loneliness described in the literature would be identifiable in participants’ stories, and if so, to further our understanding of how these facets are experienced in individual lives.

Participants’ stories provided clear examples of social, emotional and existential loneliness. Many participants described social loneliness through stories of missing friends or colleagues as a result of social distancing measures. These participants’ needs were generally different from those who described emotional loneliness through a desire for a romantic partner or close family connections: emotional loneliness appeared to be felt more inwardly, and more deeply, than social loneliness. This distinction supports the existence of separate facets of emotional and social loneliness established by Weiss (1973). Further, there was evidence of a theme of existential loneliness, where participants described feeling completely alone in the world, or expressed fear of being alone. The presence of existential loneliness in these participants’ low point stories indicates that it is not just felt by ill and dying people, which has been the focus of all known qualitative studies on existential loneliness (e.g., Mayers & Svartberg, 2001; Ettema et al., 2010). Rather, existential loneliness should be included within the common understanding of loneliness, as well as social and emotional facets.

There were also clear links in participants’ stories between loneliness and the positive experience of being alone, solitude. Solitude was described in a range of contexts, including being alone in nature, being away from the world with an intimate other, or enjoyed within a wider social context, such as a cafe, corresponding to ways of experiencing solitude briefly discussed in previous studies (e.g., Long & Averill, 2003; Lay et al., 2019), indicating that, like loneliness, solitude is multi‐faceted. Solitude contrasted particularly noticeably with the theme of existential loneliness: these facets appeared to be at the extreme ends of an aloneness continuum. This suggests that existing definitions of loneliness (e.g., van Tilburg, 2020) could be extended into a more inclusive overarching construct of aloneness, to include solitude as well as facets of loneliness.

A key difference between the facets of the loneliness constructs was the “lack” experienced. Generally, participants who reported social and emotional loneliness indicated that they desired emotional or social relationships, as suggested by Weiss (1973) and further studies on these constructs (e.g., DiTommaso & Spinner, 1997). However, existential loneliness has been described as having no remedy (Mayers & Svartberg, 2001), and this was supported in the present study by participants’ descriptions of feeling as though they were completely alone and always would be, or that they were afraid to die alone. These descriptions varied in intensity, but suggested that participants experiencing existential loneliness may need support beyond social or emotional connections. Consequently, being able to identify and distinguish between the different facets of aloneness, why and when each might lead to or result from another, and their interrelationship with poor mental health, particularly depression, is crucial to enable appropriate interventions to be developed to address them individually.

The participants in the study provided some insight into the way that facets of aloneness interact. Stories containing loneliness themes often included more than one facet. However, participants’ descriptions indicated that these overlaps did not negate the existence of distinct facets of aloneness, but that one facet might change into another, or that more than one type might co‐exist. It appeared that existential loneliness in participants’ stories may have stemmed from worsening emotional and social forms of loneliness: this contrasts with previous literature, which has suggested that existential loneliness is the basis of emotional and social loneliness (e.g., Mayers & Svartberg, 2001). Nevertheless, the rich narrative data in this study supports van Tilburg's (2020) quantitative findings of distinct social, emotional and existential loneliness types.

There were clear indications of social themes in high point stories from lonely participants, corresponding to the majority of existing loneliness studies suggesting social interventions. This included those who used social media to maintain connections with friends and family. These data indicated that social media may be useful to alleviate loneliness for those who already have meaningful connections in real life, particularly when used in the form of direct virtual interactions, for example Zoom. However, for those who are already lonely in real life, social media appeared more likely to impact negatively on their wellbeing by reminding them of their loneliness. This corresponds with previous studies on social media and loneliness (e.g., Nowland et al., 2018; López et al., 2019). This suggests that virtual social interventions in lockdowns may be beneficial, but that they do not replace real connections to counter loneliness if these were previously lacking.

Further, individuals reporting a wide range of high points appeared better protected against loneliness, suggesting that both solitude and social connections are important. Being able to enjoy a wide range of high points also appeared to correlate with increased personal agency and stability, aligning with research demonstrating that autonomy is crucial for wellbeing (e.g., Thomas & Azmitia, 2019). Consequently, the existing focus on social mechanisms to address loneliness, whilst important, may be too narrow. Given the wide range of high point themes in participants’ stories, it appears that individuals have a varying degree of aloneness requirements, ranging from social to solitary.

## IMPLICATIONS FOR PRACTICE

These findings provide a useful insight into how individual experiences of loneliness could be addressed. The UK Government had already recognized the need to recognize loneliness as a matter of public policy before the COVID‐19 pandemic, and this stance has been strengthened during the national lockdowns. For example, their recent report on social connection in the COVID‐19 crisis recommended improving short‐ and long‐term social support to isolated individuals, both in‐person (such as via support organizations) and virtually (Great Britain. All‐Party Parliamentary Group on Social Integration, 2020). However, it is proposed that rather than assuming that social mechanisms will alleviate all facets of loneliness, a broader approach should be taken by policy‐makers, particularly when there are indications of existential loneliness. This will be particularly important if and when policies to cover future lockdowns are implemented, although loneliness is a pervasive issue that will perpetuate beyond the pandemic context.

It is therefore suggested that alongside social methods, policy‐makers should consider mechanisms to encourage individuals to learn how to enjoy aloneness autonomously, whether this means being alone in nature, by oneself within a wider social setting, or being occupied in a pleasurable solitary activity. Support for nurturing and accessing such activities could be developed in the context of both health and education policy. However, the extent and appropriate level of solitude and social intervention will depend upon the individual; for some, social interventions will remain the predominant way to alleviate their loneliness. It is therefore crucial to understand individual needs and experiences of aloneness.

## LIMITATIONS AND FUTURE DIRECTIONS

Future studies of aloneness should aim to develop these findings by exploring the constructs in more depth. It would be useful to ask a larger sample of the general population to describe their experiences of aloneness, including some targeted questions, particularly about existential loneliness and solitude. There is an urgent need to understand how existential loneliness links to chronic loneliness and depression, as well as the direction of effects between existential and other forms of loneliness. It would also be beneficial to explore the different facets of solitude, and ways in which solitude might complement existing social interventions against loneliness.

The present study employed a retrospective narrative design, analyzing the themes identified within participants’ stories. Consequently, the study is exploratory and its findings are not intended to be generalized. Further, the quantitative, single‐item measure of loneliness provided by participants was developed and used to provide a fuller picture of their self‐perceptions of loneliness during the COVID‐19 lockdown alongside their narratives, and should not be considered a validated measure of loneliness. It is acknowledged that using a single‐item measure may be less robust than a multiple‐item measure, although it allowed participants to provide a straightforward answer to their holistic impression of their loneliness during the COVID‐19 lockdown.

Data on socio‐economic status and ethnic/racial background were not gathered as part of this study. In future studies, it would be useful to gather and provide a summary of this data to fully understand the background characteristics of participants.

Further, differences in participants’ experiences of local COVID‐19 lockdown restrictions have not been identified specifically during analysis. Although the study's focus was to identify the aloneness constructs themselves, and the majority of participants were from the UK, broader factors such as local lockdowns may have had an effect on the extent and type of aloneness experienced.

## CONCLUSION

The isolation imposed on much of the global population during the COVID‐19 pandemic has highlighted the need to address loneliness. The present study contributes to this research, proposing an aloneness construct encompassing social, emotional and existential loneliness and solitude. Understanding the way that these facets interact is important because each one requires different interventions. In particular, existential loneliness is recognized to entail feelings of despair and fear, which cannot simply be remedied through social connections. Consequently, solitude‐focused policies and interventions may provide a positive opposite to the most negative feelings of loneliness within the aloneness construct.

## CONFLICT OF INTEREST

The authors have no conflict of interest to declare.

## Data Availability

This data was not pre‐registered. The data used in the research are available. The data can be obtained by emailing: rowena.leary@york.ac.uk.
